# Increased nitroglycerin-mediated vasodilation in migraineurs without aura in the interictal period

**DOI:** 10.1007/s10396-018-0880-3

**Published:** 2018-05-23

**Authors:** Kazumi Fujioka, Minoru Oishi, Akira Fujioka, Tomohiro Nakayama

**Affiliations:** 1 Division of Laboratory Medicine, Department of Pathology and Microbiology, Nihon University School of Medicine, 30-1 Oyaguchi-kamicho, Itabashi-ku, Tokyo, 173-8610 Japan; 2 Department of Internal Medicine, Izutobu General Hospital, Shizuoka, Japan; 3 Fujioka Dermatological Clinic, Tokyo, Japan

**Keywords:** Migraine headache, NO sensitivity, Nitroglycerin, Flow-mediated vasodilation, Nitroglycerin-mediated vasodilation

## Abstract

**Purpose:**

Migraine is associated with vascular disorders, but the underlying mechanism is unknown. Nitric oxide (NO) sensitivity is believed to play a major role in migraine pathophysiology. We investigated flow-mediated vasodilatation (FMD) and nitroglycerin-mediated vasodilatation (NMD) of the brachial artery by means of a key molecular mediator, NO, in patients with migraine without aura in the interictal period whether the abnormality is found.

**Methods:**

A total of 12 patients with migraine without aura and 12 matched healthy controls were enrolled in this study. FMD and NMD were measured in all patients and controls using brachial artery ultrasonography.

**Results:**

There was no significant difference in brachial artery diameter between migraineurs and nonmigraineurs (3.39 ± 0.68 vs 3.89 ± 0.67 mm, respectively; *p* = 0.083). A significant difference in FMD was not found between migraineurs and nonmigraineurs (6.94 ± 5.72 vs 6.08 ± 2.98%, respectively; *p* = 0.651). However, NMD in migraineurs was significant higher than that in nonmigraineurs (21.56 ± 7.36 vs 14.23 ± 7.41%, respectively; *p* = 0.024).

**Conclusion:**

We think that patients with migraine without aura in the interictal period have selective sensitivity in dilator response to nitroglycerin and may have systemic NO sensitivity.

## Introduction

Flow-mediated vasodilation (FMD), an endothelium-dependent function, and nitroglycerin-mediated vasodilation (NMD), an endothelium-independent function, in the brachial artery is a useful tool for evaluating vascular endothelial and vascular smooth muscle cell (VSMC) function [1]. A principal mediator of FMD and NMD is endothelium-derived nitric oxide (NO) and exogenous NO from nitroglycerin (NTG). Decreased FMD [2] and increased NMD and decreased FMD [3] have been described in reports of patients with migraine without aura in the interictal period. In previous studies, when systemic NTG, an NO donor, was administrated to patients with migraine without aura in the interictal period, an increased sensitivity to NO was demonstrated [4, 5]. Furthermore, coronary spastic angina and Raynaud’s phenomenon, have been described in association with migraineurs [6]. There are a few reports of the underlying mechanism in patients with migraine that evaluated endothelial and VSMC effects [3, 7, 8]. Vascular tone change in migraineurs with or without aura [9], NO sensitivity with endothelial dysfunction using NTG [3], and VSMC sensitivity to NO without endothelial dysfunction using nitroprusside (NP) [7, 8] in migraineurs without aura have been reported with respect to migraine pathophysiology. Napoli et al. have noted VSMC dysfunction in patients with migraine using NP, a vasodilator directly acting on VSMC [7, 8]. Though NTG and NP act directly at the level of the arterial smooth muscle cell and produce an endothelium-independent dilatation response, different reactions between NTG and NP have been recognized in migraineurs without aura in the interictal phase. We speculate that migraineurs without aura may have a systemic vascular abnormality, as described in previous reports [2, 3, 6]. We suggest that patients with migraine without aura in the interictal period have selective sensitivity in dilator response to NTG and may have systemic NO sensitivity to NTG.

## Materials and methods

### Study population

Twelve patients who fulfilled the diagnostic criteria of migraine without aura were enrolled in the study between January 2008 and May 2014. The study was performed in 12 patients with migraine and 12 healthy subjects who served as controls (Table 1). The controls were recruited from the hospital and were matched to the patients with regard to age and sex. The diagnosis of migraine was made by an expert in migraine-related neurology, according to the criteria of the International Headache Society [10, 11]. All vasoactive medications were withheld for at least a few days. On the day of the study, patients had been free from migraine attacks for at least 5 days. Female patients were allowed to undergo ultrasonographic evaluation either in the luteal or follicular phase. For the FMD examination, patients fasted for 12 h before the study and they were studied in a quiet, temperature-controlled room.

**Table 1:** Baseline characteristics of migraineurs and nonmigraineurs (mean ± SD)

	Migraineurs	Nonmigraineurs	*p* value
Age, years	45.3 ± 11.4	52.1 ± 12.0	0.167
Heart rate beats/min	66.2 ± 10.6	66.5 ± 8.5	0.933
SBP, mmHg	123.6 ± 12.2	137.1 ± 27.0	0.161
DBP, mmHg	80.4 ± 12.8	80.6 ± 12.7	0.973
TC, mg/dL	205.7 ± 28.6	224.2 ± 31.6	0.147
TG, mg/dL	83.3 ± 29.2	121.3 ± 61.8	0.067
BMI, kg/m^2^	21.4 ± 3.0	24.6 ± 4.8	0.062

*SBP* systolic blood pressure, *DBP* diastolic blood pressure, *BMI* body mass index

### Vascular reactivity

FMD of the brachial artery was determined using a high-resolution B-mode ultrasonographic system (UNEXEF 18G, UNEX, Nagoya, Japan) with a linear transducer with a mid frequency of 7.5 MHz, using the technique described in a previous report [1]. Briefly, a pneumatic tourniquet placed around the forearm was inflated to elevate pressure 50 mmHg more than systolic blood pressure, followed by deflation after 5 min. The scan was performed using autocalculation. Fifteen minutes were then allowed for vessel recovery, and a further scan at rest was then recorded. An exogenous NO donor, such as a single high dose (0.3 mg) of NTG spray, was given to determine the maximum obtainable vasodilator response, and to serve as a measure of endothelium-independent vasodilation reflecting VSMC function, in accordance with a previous report [1]. FMD and NMD were expressed as the change in the post-stimulus (flow and nitrate mediated) diameter as a percentage of the baseline diameter. FMD in mm represents absolute change in the post-flow stimulus. Post-nitroglycerin brachial artery diameter (P-NTGD) shows the maximal brachial artery diameter after administration of NTG [12]. Absolute NMD represents absolute change in the post-nitrate-mediated stimulus. All measurements were performed during a pain-free period in migraineurs without aura.

### Carotid ultrasonography

The intima-media thickness (IMT) of the bilateral common carotid arteries (CCA) was measured by ultrasonography with a 10-MHz probe using an ultrasound system (Aplio SSA-700A, Toshiba Medical Systems, Tochigi, Japan). IMT measurements were made on the visible CCA in either the near or the far wall, and the maximum IMT was defined as IMT-C max. Measurements of IMT were made within a region free of plaque according to the established consensus [13].

### Brachial-ankle pulse wave velocity (baPWV) measurement

baPWV was measured using a volume-plethysmographic apparatus (form PWV/ABI, Colin, Komaki, Japan), and ankle brachial pressure index (ABI), which is useful for detecting peripheral artery disease, was measured simultaneously with these machines in accordance with a described method [14].

### Statistical analysis

Numerical variables were expressed as mean ± SD. For categorical data, Mann–Whitney *U* test was used. Statistical significance was defined as a *p* value of less than 0.05. The statistical analyses were performed using the SPSS software package (version 16.0; SPSS Inc., Chicago, IL).

## Results

**Fig. 1:**
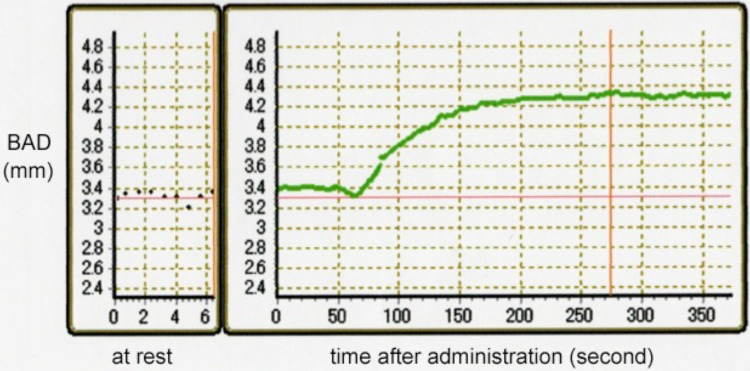
Representative time course of NMD response in migraineurs

**Fig. 2:**
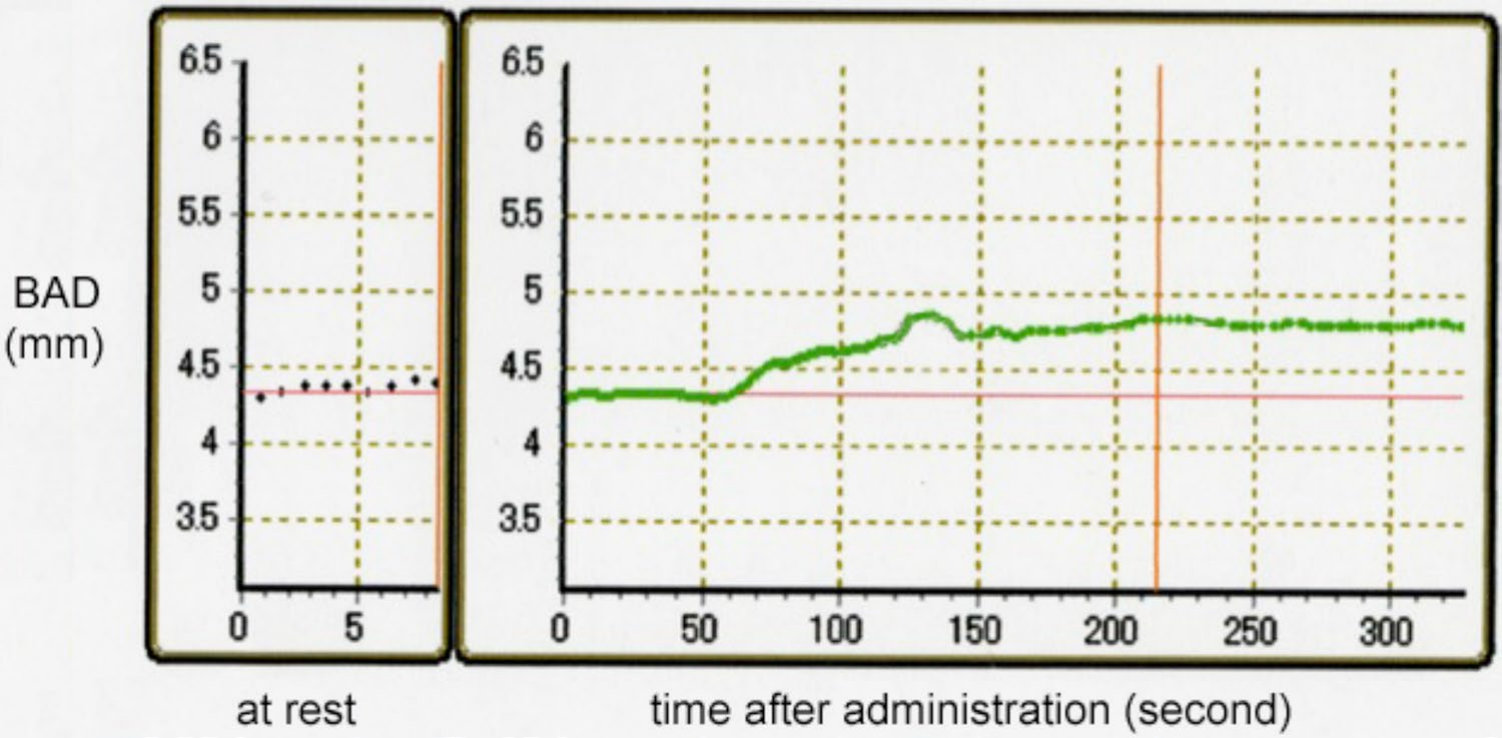
Typical time course of NMD reaction in nonmigraineurs

Baseline characteristics of the study population are shown in Table 1. Mean age of the patients was 45.3 ± 11.4 years in migraineurs (range 19–62 years; 10 females, 2 males) and 52.1 ± 12.0 years in nonmigraineurs (range 25–66 years; 10 females, 2 males). Brachial artery measurements of migraineurs and nonmigraineurs are presented in Table 2. There was no significant difference in brachial artery diameter (BAD) between migraineurs and nonmigraineurs (3.39 ± 0.68 vs 3.89 ± 0.67 mm, respectively; *p* = 0.083). FMD examination showed no significant difference between migraineurs and nonmigraineurs (6.94 ± 5.72 vs 6.08 ± 2.98%, respectively; *p* = 0.651). However, NMD in migraineurs was significantly higher than that in nonmigraineurs (21.56 ± 7.36 vs 14.23 ± 7.41%, respectively; *p* = 0.024). Figure 1 shows the representative time course of NMD response in migraineurs, and Fig. 2 shows NMD reaction in nonmigraineurs. Migraine-like delayed headache after administration of NTG did not occur in migraineurs, and delayed headache did not appear in nonmigraineurs. Structural and mechanical arterial wall properties of migraineurs and nonmigraineurs are presented in Table 3. There was no significant difference between migraineurs and nonmigraineurs in relation to both right and left IMT-Cmax, baPWV, and ABI.

**Table 2:** Brachial artery measures (mean ± SD)

	Migraineurs	Nonmigraineurs	*p* value
BAD-base, mm	3.39 ± 0.68	3.89 ± 0.67	0.083
BAD-max, mm	3.60 ± 0.61	4.12 ± 0.66	0.059
FMD, mm	0.21 ± 0.16	0.23 ± 0.12	0.748
FMD, %	6.94 ± 5.72	6.08 ± 2.98	0.651
NMD, %	21.56 ± 7.36	14.23 ± 7.41	0.024
Absolute NMD, mm	0.72 ± 0.23	0.54 ± 0.24	0.073
P-NTGD, mm	4.15 ± 0.62	4.54 ± 0.58	0.124
FMD/NMD ratio	0.36 ± 0.28	0.45 ± 0.23	0.496

*BAD* brachial artery diameter, *BAD-base* BAD baseline diameter, *BAD-max* BAD maximal diameter, *FMD* flow-mediated vasodilation, *NMD* nitroglycerin-mediated vasodilation, *P-NTGD* post-nitroglycerin brachial artery diameter

**Table 3:** Structural and mechanical arterial wall properties (mean ± SD)

	Migraineurs	Nonmigraineurs	*p* value
Rt IMT-Cmax, mm	0.71 ± 0.23	0.88 ± 0.39	0.339
Lt IMT-Cmax, mm	0.81 ± 0.26	0.80 ± 0.18	0.899
Rt baPWV, cm/s	1276.9 ± 103.4	1471.8 ± 344.6	0.119
Lt baPWV, cm/s	1296.2 ± 96.5	1466.9 ± 336.2	0.158
Rt ABI	1.13 ± 0.05	1.14 ± 0.07	0.711
Lt ABI	1.11 ± 0.05	1.13 ± 0.08	0.542

*Rt* right, *Lt* left, *IMT-Cmax* maximum intima-media thickness of common carotid artery, *baPWV* brachial-ankle pulse wave velocity, *ABI* ankle brachial pressure index

## Discussion

FMD and NMD examinations of the brachial artery are useful procedures for estimating vascular endothelial and VSMC function by means of a key molecular mediator, NO. Increased NMD and decreased FMD in the brachial artery were observed in patients with migraine without aura in the interictal period [3]. It has been reported that migraineurs are supersensitive to both exogenous NO from NTG and endogenous NO formed in the cerebral arterial endothelium [4]. In previous studies, when systemic NTG, an NO donor, was administrated to patients with migraine without aura in the interictal period, an increased sensitivity to NO was demonstrated [4, 5].

There have been some reports of FMD examinations in patient with migraine, which found reduced FMD [2, 3, 9], normal FMD [15, 16], and increased FMD [17].

With respect to increased NMD, there is only one study in patients with migraine without aura in the interictal phase in the literature [3]. There have been a few reports with regards to decreased NMD studies including cardiovascular disease (CVD) [18], type 2 diabetic patients (DM) [19], and obesity [20]. NMD is affected or determined by VSMC dysfunction and the surrounding extracellular matrix in the medial layer of the arterial wall. The accelerating risk factors for atherosclerosis, such as hyperglycemia, dyslipidemia, hypertension, and uremia, may exert long-term cumulative effects on VSMC and the surrounding matrix in the medial layer rather than on the endothelium [18]. As a result, reduced NMD has been reported in patients with CVD, DM, and obesity [18–20].

Yetkin et al. [3] reported decreased FMD and increased NMD in patients with migraine without aura in the interictal period. They suggested that the baseline endothelial dysfunction may underlie the pathogenesis of the increased NMD due to a deficiency in endothelial nitric oxide bioactivity, as described in a previous report [21]. Kugiyama et al. reported a patient with coronary spastic angina who showed selective sensitivity in dilator response to NTG compared with atrial natriuretic peptide, as a nitrovasodilator, suggesting those possible mechanisms caused by the enhancement of soluble guanylate cyclase activity and/or conversion of NO bioactivity from NTG or an increase in cyclic guanosine monophosphate (cGMP) activity in the effector system of smooth muscle cells [21].

Napoli et al. explored endothelial and VSMC components of vascular reactivity by plethysmography measurement of forearm blood flow during infusion of vasoactive agents including acetylcholine (Ach) and NP, a vasodilator directly acting on VSMC, into the brachial artery [7, 8]. The forearm NO and cGMP balance during intrabrachial Ach infusion was examined, and production of NO and cGMP was also quantified [7]. They concluded that the patients with migraine in the interictal period had a reduced sensitivity of their VSMCs to the NO released by endothelial cells [7]. In contrast, the response to NO showed a restored sensitivity of VSMCs during headache attack [8].

In our study, the increased NMD result agreed with that in the previous report [3], but it was different from that in Napoli’s report [7, 8] using NP, a vasodilator with a direct action on VSMC. As NTG and NP act directly at the level of the arterial smooth muscle cell and produce an endothelium-independent dilatation response, a different reaction has been recognized in migraineurs without aura in the interictal phase. Therefore, we speculate that NO reactivity to NTG compared with other NO donors is a selective phenomenon in patients with migraine. Patients with migraine may have NO sensitivity to NTG both in the cerebral arteries and systemic arteries. Because it has been noted that the brachial artery reflects systemic vessel status, we also suggest that the mechanism underlying migraine may be diffuse vascular vasomotion abnormalities to NTG, as reported in previous studies [2, 3, 6].

Vanmolkot et al. indicated that a smaller diameter of muscular artery including the brachial artery and decreased compliance are associated with a generalized increase in vascular smooth muscle tone in patients with migraine with or without aura [9]. They speculated that an increased arterial tone predisposed patients with migraine for vasospastic disorders, including variant angina and Raynaud’s phenomenon [9]. On the basis of a previous report [9], a smaller BAD reflects an increased arterial tone. Our data showed that a smaller BAD in patients with migraine compared with controls without significant difference tended to have a vascular tone abnormality, as previously described [9].

Alternatively, migraine is associated with cardiovascular disorders, but the underlying mechanism is unknown. Arterial structure and function are important determinants of cardiovascular morbidity and mortality. Evaluation of morphology and functional arterial properties such as carotid IMT, PWV, and arterial stiffness has also been reported [9]. We assessed differences between patients with migraine and controls. We found no significant differences between patients and controls in terms of carotid IMT-Cmax, baPWV, and ABI.

We also investigated a biological marker, von Willebrand factor (vWF), an indicator of serum endothelial function [22]. Like FMD, a significant difference in vWF was not found between patients with migraine and controls (77.92 ± 45.73 vs 95.83 ± 27.93%, respectively; *p* = 0.259). Our result indicates that patients with migraine without aura are not predisposed to cardiovascular disorders, even though our sample size was small. Our FMD result was different from that in Yetkin’ s study [3]. Therefore, our data indicate that endothelial dysfunction may not underlie the pathogenesis of increased NMD, as previously reported [3], and that the sensitive dilator response to NTG is due to a VSMC abnormality in migraineurs without aura.

FMD and NMD studies have been established as noninvasive and reliable procedures for evaluating endothelial and VSMC function. Our increased NMD value agreed with that in the previous report [3], but it was different from that in Napoli’s report [7, 8] using NP, a vasodilator with a direct action on VSMC. NTG may be a selective agent in patients with migraine. We think that NO sensitivity to NTG in patients with migraine without aura is a specific and selective response, and that patients with migraine may have systemic NO sensitivity to NTG.

## Conclusion

We believe that patients with migraine without aura in the interictal period have a selective sensitivity in dilator response to NTG and may have systemic NO sensitivity to NTG.
